# Supplementary material for: Plastid thylakoid architecture optimizes photosynthesis in diatoms
Source: Nat Commun. 2017 Jun 20;8:15885. doi: 10.1038/ncomms15885 (PMC5481826; doi:10.1038/ncomms15885)
Supplement: Supplementary Information [file ncomms15885-s1.pdf]

Type of file: PDF
Size of file: 0 KB
Title of file for HTML: Supplementary Information
Description: Supplementary Figures, Supplementary Tables and Supplementary Note

Type of file: MP4
Size of file: 0 KB
Title of file for HTML: Supplementary Movie 1
Description: Focus Ion Beam Scanning Electron Microscopy (FIB-SEM) based 3D reconstruction of a whole cell of *P. tricornutum* and 3D structure of the chloroplast (green) mitochondrion (red) and nucleus (blue) in *P. tricornutum* cells. 1 Voxel: 4 nm.

Type of file: MP4
Size of file: 0 KB
Title of file for HTML: Supplementary Movie 2
Description: High resolution Focus Ion Beam Scanning Electron Microscopy (FIB-SEM) of a whole cell of *P. tricornutum*. 1 voxel: 2 nm.

Type of file: PDF
Size of file: 0 KB
Title of file for HTML: Peer Review File
Description:

Supplementary information

Supplementary Note 1: Output results of the Logistic regression from the R software.

Original model: Complex = CORE + PERIPHERAL + ENVELOP + PYRENOID

```
  summary(glm(COMPLEX~CORE+PERIPHERAL+ENVELOP+PYRENOID,binomial))

Call:
glm(formula = COMPLEX ~ CORE + PERIPHERAL + ENVELOP + PYRENOID,
    family = binomial)

Deviance Residuals:
    Min       1Q   Median       3Q      Max
-2.5568  -0.6790  -0.1991   0.6311   2.8584

Coefficients: (1 not defined because of singularities)
            Estimate Std. Error z value Pr(>|z|)
(Intercept)  -0.2142     0.8657  -0.247  0.80461
CORE          3.4439     1.0625   3.241  0.00119 **
PERIPHERAL   -4.8898     1.1578  -4.223 2.41e-05 ***
ENVELOP       2.3609     1.8486   1.277  0.20155
PYRENOID          NA         NA      NA       NA
---
Signif. codes:  0 '***' 0.001 '**' 0.01 '*' 0.05 '.' 0.1 ' ' 1

(Dispersion parameter for binomial family taken to be 1)

    Null deviance: 293.42  on 211  degrees of freedom
Residual deviance: 186.84  on 208  degrees of freedom
AIC: 194.84

Number of Fisher Scoring iterations: 5
```

=> NA is give as a coefficient in the regression because that the variable in question is linearly related to the other variables. Consequence of the normalization of the count table.

Second model: Complex = CORE + PERIPHERAL + ENVELOP

```
> summary(glm(COMPLEX~PERIPHERAL+CORE+ENVELOP,binomial))

Call:
glm(formula = COMPLEX ~ PERIPHERAL + CORE + ENVELOP, family = binomial)

Deviance Residuals:
    Min       1Q   Median       3Q      Max
-2.5568  -0.6790  -0.1991   0.6311   2.8584

Coefficients:
            Estimate Std. Error z value Pr(>|z|)
(Intercept)  -0.2142     0.8657  -0.247  0.80461
PERIPHERAL   -4.8898     1.1578  -4.223 2.41e-05 ***
CORE          3.4439     1.0625   3.241  0.00119 **
ENVELOP       2.3609     1.8486   1.277  0.20155
---
Signif. codes:  0 '***' 0.001 '**' 0.01 '*' 0.05 '.' 0.1 ' ' 1

(Dispersion parameter for binomial family taken to be 1)

    Null deviance: 293.42  on 211  degrees of freedom
Residual deviance: 186.84  on 208  degrees of freedom
AIC: 194.84

Number of Fisher Scoring iterations: 5
```

Final model: Antibody = CORE + PERIPHERAL

```
glm2 <- glm(COMPLEX~PERIPHERAL+CORE,binomial)
> glm1 <- glm(COMPLEX~PERIPHERAL+CORE+ENVELOP,binomial)

> anova(glm1,glm2,test="Chisq")
Analysis of Deviance Table

Model 1: COMPLEX ~ PERIPHERAL + CORE + ENVELOP
Model 2: COMPLEX ~ PERIPHERAL + CORE
  Resid. Df Resid. Dev Df Deviance Pr(>Chi)
1       208     186.84
2       209     188.52 -1  -1.6768   0.1954


> summary(glm2)

Call:
glm(formula = COMPLEX ~ PERIPHERAL + CORE, family = binomial)

Deviance Residuals:
    Min       1Q   Median       3Q      Max
-2.5627  -0.7080  -0.2039   0.6151   2.9218

Coefficients:
            Estimate Std. Error z value Pr(>|z|)
(Intercept)   0.5223     0.6444   0.810  0.41767
PERIPHERAL   -5.5727     1.0373  -5.372 7.77e-08 ***
CORE          2.7233     0.8839   3.081  0.00206 **
---
Signif. codes:  0 ‘***’ 0.001 ‘**’ 0.01 ‘*’ 0.05 ‘.’ 0.1 ‘ ’ 1

(Dispersion parameter for binomial family taken to be 1)

    Null deviance: 293.42  on 211  degrees of freedom
Residual deviance: 188.52  on 209  degrees of freedom
AIC: 194.52

Number of Fisher Scoring iterations: 5

> drop1(glm2,test="Chisq")
Single term deletions

Model:
COMPLEX ~ PERIPHERAL + CORE
           Df Deviance    AIC    LRT  Pr(>Chi)
<none>          188.52 194.52
PERIPHERAL  1   222.98 226.98 34.462 4.347e-09 ***
CORE        1   198.25 202.25  9.733   0.00181 **
---
Signif. codes:  0 ‘***’ 0.001 ‘**’ 0.01 ‘*’ 0.05 ‘.’ 0.1 ‘ ’ 1
```

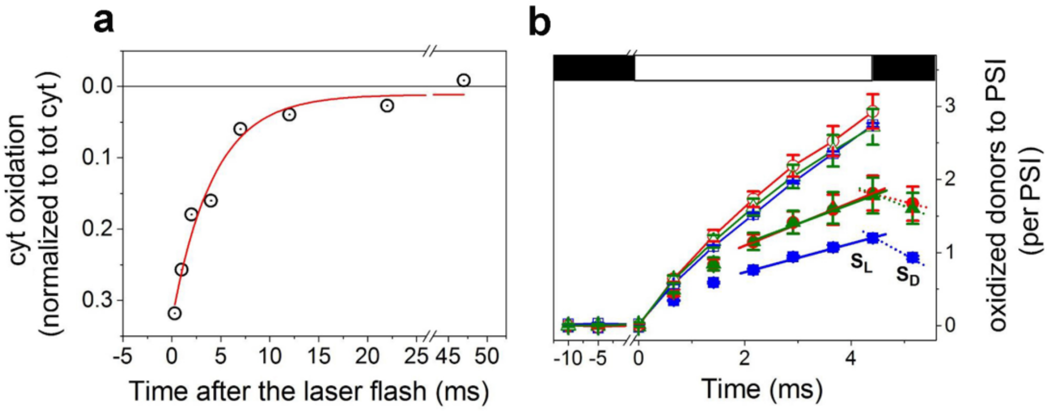

Supplementary Figure 1: PSI/cytochrome stoichiometry and deconvolution of PSI oxidation kinetics in *P. tricornutum*. (**a**) Cyt /PSI stoichiometry. Cells were exposed to a saturating single turnover laser flash to generate 1 turnover per PSI and the amount of oxidized c-type cytochrome was calculated 300 µs after the flash (i.e. when P700 is fully re-reduced by the cytochromes) This amount was normalized to the total amount of cyt oxidized in continuous light in the presence of DCMU (40 µM). Because the flash oxidizes 33% of the cyt c type cytochromes, we conclude there are ~3 c-type cytochromes (cyt c_6 + cyt *f*) per PSI. (**b**) Procedure employed to evaluate the rates of PSI oxidation in the light in the case of the total donors to PSI pool. Open bar: light on. Closed bar: light off. The slope measured after the light is switched off (S_D) allows calculating the dark re-reduction rates of the PSI donor pool in the control (closed blue squares), DCMU treated samples (closed red circles) and DCMU + HA treated samples (closed green triangles). The sum of this rate plus the apparent oxidation rate in the light (S_L) provides the real rate of oxidation of the total donors to PSI oxidation kinetics, (open symbols). See methods for further description.

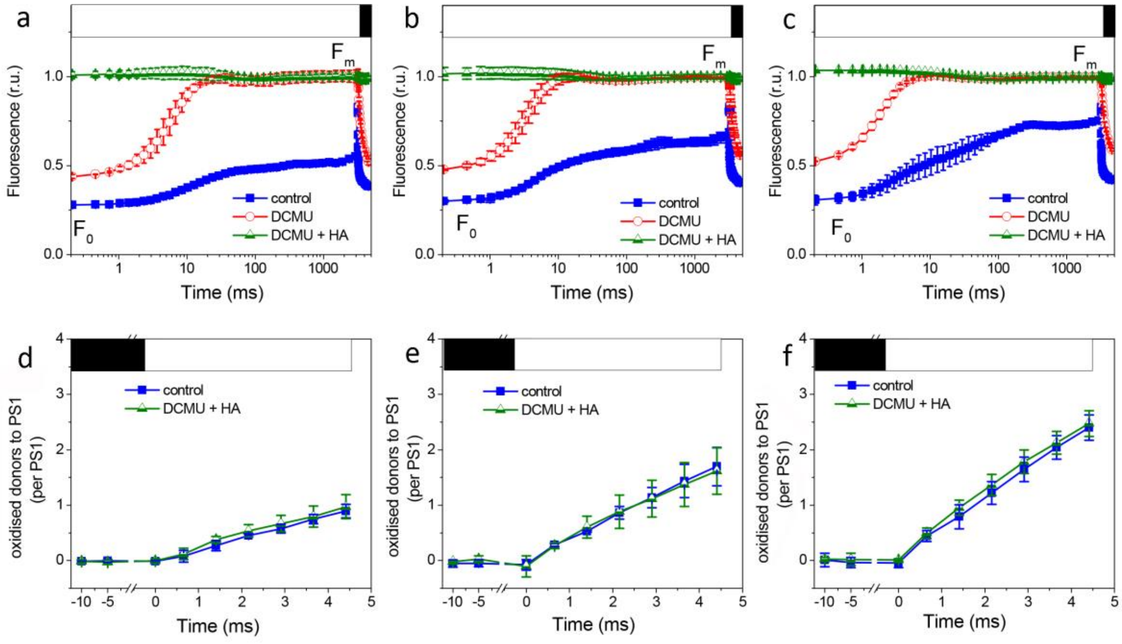

Supplementary Figure 2: Evaluation of light energy spillover in *P. tricornutum* cells exposed to different light intensities. (**a-c**) Fluorescence emission kinetics in control (blue squares), DCMU (40 µM) poisoned cells (red circles) and DCMU + HA (2 mM) poisoned cells (green triangles) upon exposure to 150 µm photons m^{-2} s^{-1} (**a**) 300 µm photons m^{-2} s^{-1} (**b**) and 590 µm photons m^{-2} s^{-1} (**c**). (**d-f**) Kinetics of oxidation of the entire pool of PSI electron donors at a light intensity of 150 µm photons m^{-2} s^{-1} (**d**), 300 µm photons m^{-2} s^{-1} (**e**) and 590 µm photons m^{-2} s^{-1} (**f**). Mean ± SEM (n = 6). F_0: mimimum fluorescence emission (active PSI). Fm: maximum fluorescence emission (inactive PSI). Closed bar: actinic light off. Open bar: actinic light on.

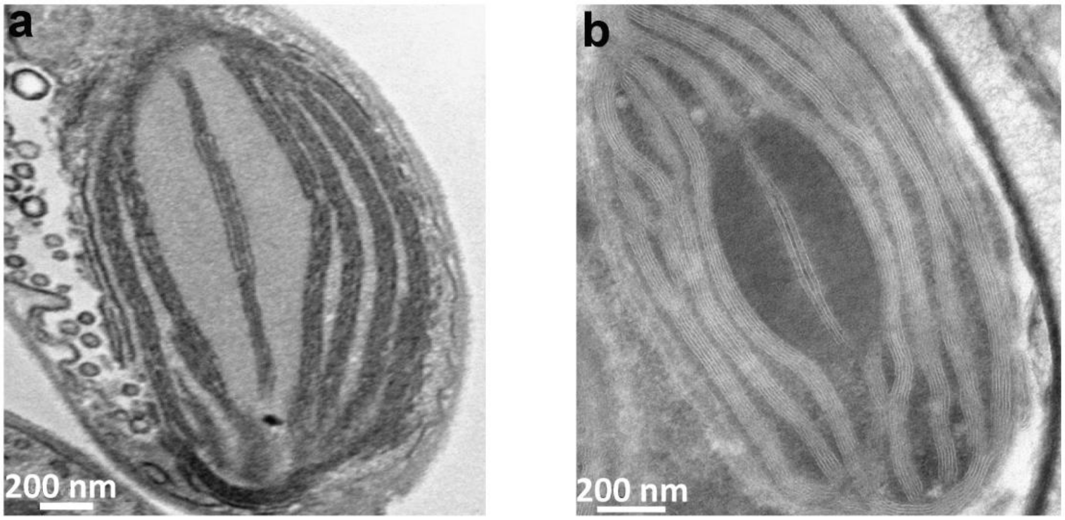

Supplementary Figure 3: Tokuyasu cryosection enhances the resolution of EM pictures of *P. tricornutum* thylakoid membranes. EM images of *P. tricornutum* from a sample fixed in resin (**a**) and a sample prepared using the Tokuyasu cryosection (**b**). Bar: 200 nm.

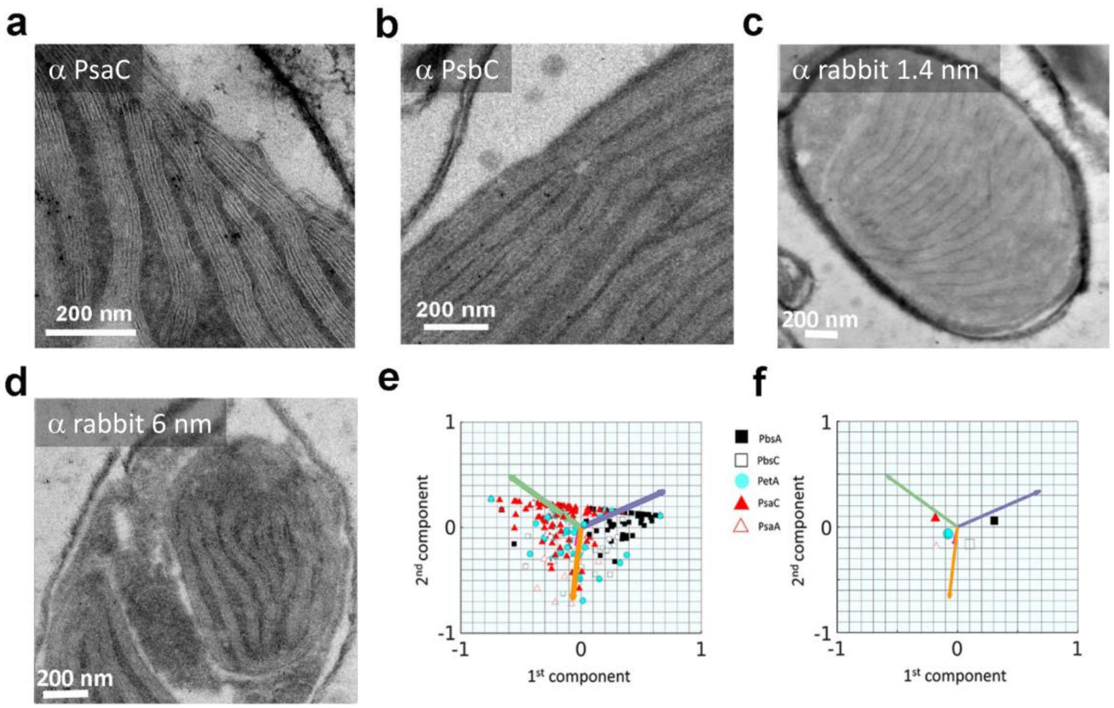

Supplementary Figure 4. Immunolocalisation of PSI, and PSII in the thylakoid membranes of *P. tricornutum*. (a) Localisation of PSI using an antibody against the PsaC subunit. (b) Localisation of PSI using an antibody against the PsbC subunit. (c-d) lack of immunolabelling in cells treated with the anti-rabbit secondary antibodies alone. The beads size was 1.4 nm and 6 nm, respectively. The 6 nm gold conjugate goat anti-rabbit secondary antibodies (Aurion, Wageningen NL) were used to detect PsaA and PetA. Goat anti-rabbit gold ultra-small 1.4 nm secondary antibodies (Aurion, Wageningen NL), were used to dectect PsbA, PsaC and PsbC and sections were enhanced with silver (Aurion R-Gent SE-EM) for 25 min and again washed on deionized water (6 times for 2 min). (e-f) Principal component analysis of PSI, cyt b_6f and PSI immunolocalisation with the PsbA (solid squares), PsbC (open squares), PetA (cyan circle), PsaC (solid triangles) PsaA (open triangles) antibodies. 258 images from four independent cultures were analysed. In (e) the distribution of the 5 barycentres of the different antibody distributions is presented. The point size along an axis is proportional to the standard deviation along the corresponding component. Green arrow: peripheral variable. Violet arrow: core variable. Orange arrow: pyrenoid variable. Magenta arrow: envelope variable. Bars: 200 nm.

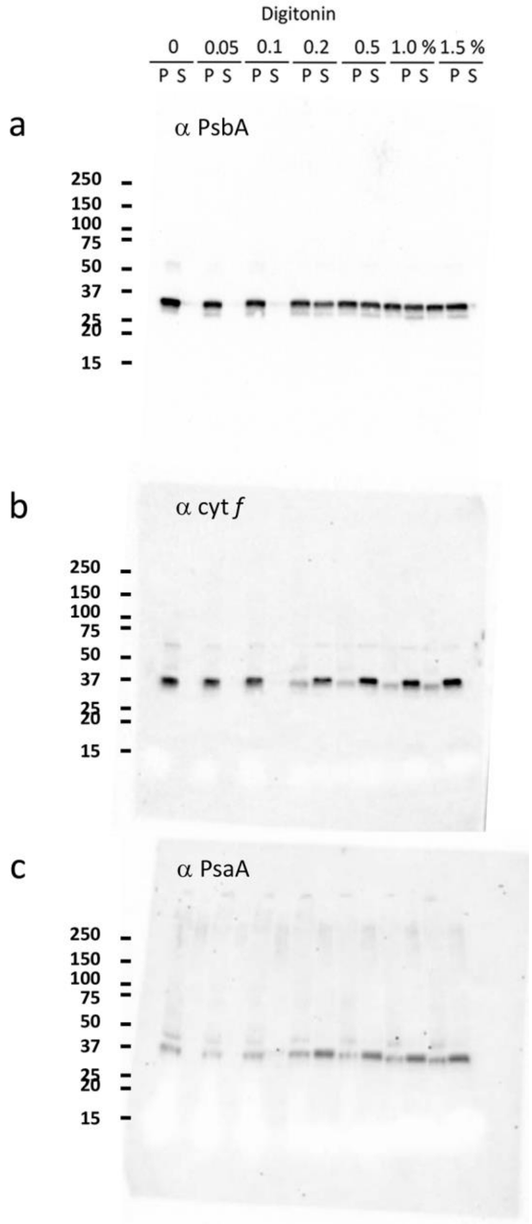

Supplementary Fig. 5 Solubilisation of *P. tricornutum* thylakoid membranes with increasing concentrations of digitonin (0, 0.05%, 0.1%, 0.2%, 0.5%, 1%, 1.5%). Pellet (P) and supernatant (S) were analysed by western blotting with the same anti PSII (a), PSII (b) and cyt b_6f antibodies (c) as in Fig. 2 a-c. Representative dataset of an experiment replicated on three different biological samples.

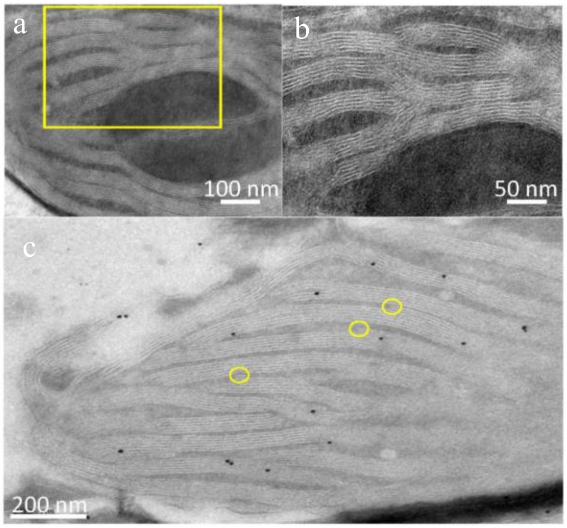

Supplementary Figure 6: Peculiar structural features of thylakoid membranes in *P. tricornutum* cells prepared with Tokuyasu cryosections. (**a**) EM images of *P. tricornutum* using the Tokuyasu cryosections reveal the existence of crosspoints between the thylakoid layers. (**b**) Magnification of the thylakoid layers intersections in the region indicated by the yellow box in panel a. (**c**) "Truncated" thylakoid membranes are observed in *P. tricornutum* cells prepared with the Tokuyasu technique. Yellow circles highlight thylakoid layers that comprise membranes that abruptly disappear, suggesting the existence of 3D interconnection between different layers of thylakoids. Ummunolabelling in panel (c) was done using antibodies against the PsbA subunit of PSI.

	Comp. 1	Comp. 2	Comp. 3	Comp. 4
**Standard deviation**	0.3277272	0.2224697	0.12278013	2.842791e-09
**Proportion of Variance**	0.6245470	0.2877940	0.08765898	4.699267e-17
**Cumulative Proportion**	0.6245470	0.9123410	1	1

Supplementary Table 1: Principal component analysis results. The first two components represent more than 91% of the variance.

	CORE	PERIPHERAL	ENVELOP	PYRENOID
**CORE**	1.00000000	-0.6557506	-0.28775490	-0.40284092
**PERIPHERAL**	-0.6557506	1.000000000	-0.17556598	-0.30080423
**ENVELOP**	0.20063404	-0.17556598	1.000000000	0.04098612
**PYRENOID**	-0.40284092	-0.30080423	0.04098612	1.00000000

Supplementary Table 2: Correlation table of the number of immunolabelling in the four possible localisations.
